# Centrality angle is a novel nephrometry score to predict tumor complexity and perioperative outcomes for partial nephrectomy

**DOI:** 10.1038/s41598-024-55448-0

**Published:** 2024-02-27

**Authors:** Shunsuke Miyamoto, Keisuke Goto, Ryo Tasaka, Yuki Kohada, Takafumi Fukushima, Kenshiro Takemoto, Takashi Babasaki, Kohei Kobatake, Yohei Sekino, Hiroyuki Kitano, Kenichiro Ikeda, Keisuke Hieda, Tetsutaro Hayashi, Nobuyuki Hinata

**Affiliations:** Department of Urology, Graduate School of Biomedical and Health Sciences, Hiroshima University, 1-2-3 Kasumi, Minami-ku, Hiroshima, 734-8551 Japan

**Keywords:** Cancer, Urology, Kidney, Cancer imaging, Renal cancer

## Abstract

To propose the centrality angle (C-angle) as a novel simple nephrometry score for the evaluation of tumor complexity and prediction of perioperative outcomes in nephron-sparing surgery (NSS) for renal tumors. The analysis was based on 174 patients who underwent robot-assisted partial nephrectomy retrospectively. C-angle was defined as the angle occupied by the tumor from the center of the kidney in the coronal CT images. Other nephrometry scores were calculated and compared with C-angle. Associations between C-angle and perioperative outcomes were examined. Significant differences were found in C-angle between tumors greater and less than 4 cm, exophytic and endophytic tumors, and hilar and non-hilar tumors. C-angle was correlated with other nephrometry scores, including RENAL, PADUA, and C-index. Significant positive correlations with WIT, operation time, and EBL, and significant negative correlations with preserved eGFR. C-angle could predict perioperative complications. Patients with a C-angle > 45° had worse perioperative outcomes, including longer operative time, longer WIT, lower rate of preserved eGFR, and complications. C-angle can be used to evaluate the complexity of renal tumors and predict perioperative outcomes. C-angle can potentially be used for decision-making in the treatment of patients and to guide surgical planning of NSS.

## Introduction

Nephron-sparing surgery (NSS) for renal tumors has been the standard procedure for tumors of less than 4 cm in diameter and an option for tumors 4–7 cm in diameter. As the indications for NSS have been carefully expanded to include complex tumors, the number of NSS operations has been increasing along with the demand for significantly more precise surgical techniques^1^. To assess the potential morbidity of surgery, nephrometry scores are used to determine the indication. The European Association of Urology (EAU) guidelines on renal cell carcinoma suggest that nephrometry scores should be used to objectively predict the potential morbidity of NSS for renal masses^2^.

Nephrometry scores can be dichotomized into two groups: a visual anatomical assessment-based nephrometry score and a mathematical assessment-based nephrometry score^3^. In 2009, the radius-exophytic/endophytic-nearness-anterior/posterior-location relative to polar lines (RENAL) score and preoperative aspects and dimensions used for an anatomical classification (PADUA) score, which were based on visual anatomical assessment, were established as common standards for the anatomical complexity of renal tumors^4,5^. These nephrometry scores have been the most reliable and popular to estimate possible morbidities and prediction of morbidity after NSS^6–9^. Meanwhile, mathematical assessment-based nephrometry scores, including the centrality index (C-index), the tumor contact surface area (CSA), and the zero ischemia index (ZII), were established as a single continuous variable score. Although useful for statistical analysis, these scoring systems are less common because of their complicated procedures and difficult reproducibility^3,10–13^. Therefore, the establishment of a simple nephrometry score that can predict perioperative outcomes with simple measurement methods would contribute to more effective preoperative evaluation when performing NSS.

In the present study, we propose the centrality angle (C-angle) as a novel, simple mathematical assessment-based nephrometry score. C-angle was measurable using only coronal CT imaging, and—was defined as the angle from center of kidney to both outlines of the tumor invading the kidney. First, we estimated C-angle in patients with a small renal mass and compared it with other nephrometry scores, including RENAL, PADUA, and C-index, to evaluate the utility of C-angle as a preoperative parameter. We also evaluated the usefulness of C-angle for predicting perioperative outcomes, including operative parameters, complications, and postoperative preserved renal function. Finally, in our cohort, C-angle was capable of predicting perioperative outcomes as effectively as other nephrometry scores.

## Materials and methods

This was a retrospective observational study conducted in accordance with the ethical standards described in the Declaration of Helsinki. This study was approved by the Research Ethics Committee of Hiroshima University and the requirement for informed consent was waived by the Research Ethics Committee of Hiroshima University (authorization number: E2016-0588).

### Patient selection

We reviewed 177 patients who underwent NSS with thin-slice preoperative CT images available at the Hiroshima University Hospital from January 2015 to May 2022. Because vertex of the C-angle and tumor was overlap, three of the 177 patients had not measurable and be excluded. Therefore, 174 patients were included in the study cohort. In all cases, 4 experienced surgeons performed robot-assisted partial nephrectomy (RAPN). Preoperative demographics (age, body mass index, and sex), tumor characteristics (tumor location, tumor size, clinical T stage, exophytic rate, and hilar tumor), nephrometry scores (C-angle, RENAL, PADUA, and C-index), and pathological features were obtained from the medical records and are listed in Table 1.

**Table 1:** Characteristics of patients, tumors and outcomes.

	95% CI
	n = 174
Patient demographics	
Age, years, median (range)	62 (26–88)
Body mass index, kg/m^2^, mean ± SD	24.2 ± 4.3
Sex, n (%)	
Male	127 (73.0)
Female	47 (27.0)
Tumor characteristics	
Kidney, n (%)	
Right	93 (53.5)
Left	81 (46.5)
Tumor size, mm, mean ± SD	26.5 ± 10.5
Clinical T stage, n (%)	
T1a	151 (86.8)
T1b	23 (13.2)
Endophytic rate, n (%)	
< 50% endophytic	56 (32.2)
50–100% endophytic	92 (52.9)
100% endophytic	26 (14.9)
Hilar tumor, n (%)	28 (23.2)
Nephrometry scores	
C-angle, °, mean ± SD	52.4 ± 27.2
C-Index score, mean ± SD	3.05 ± 1.44
RENAL score, median (range)	7 (4–10)
PADUA score, median (range)	8 (6–12)
Pathological outcome	
Malignant, n (%)	163 (93.7)
Clear	134 (77.0)
Non-clear	29 (21.6)
Non-malignant	11 (6.3)
Perioperative outcome	
Operative time, min, mean ± SD	215 ± 48
Warm ischemia time, min, mean ± SD	18.1 ± 7.4
Estimate blood loss, ml, mean ± SD	142 ± 223
Complications: Clavien–Dindo I–III, (%)	49 (28.2)
Complications: Clavien–Dindo III, (%)	8 (4.6)
Renal function	
Preoperative eGFR, ml/min/1.73 m^2^, mean ± SD	71.4 ± 16.5
Preserved eGFR (6-mo/pre), %, mean ± SD	93.5 ± 10.4

Postoperative complications were retrospectively collected through chart review by a medical doctor at 90 days after surgery according to the EAU Guidelines Panel recommendations on reporting and grading complications^14^. Postoperative complications were graded according to the Clavien–Dindo system^15^. Renal function was assessed by eGFR using serum creatinine level based on the Modification of Diet in Renal Disease equation. Postoperative renal function was defined as eGFR measured 6 months after RAPN^16^.

### Measurement method of C-angle

**Figure 1:**
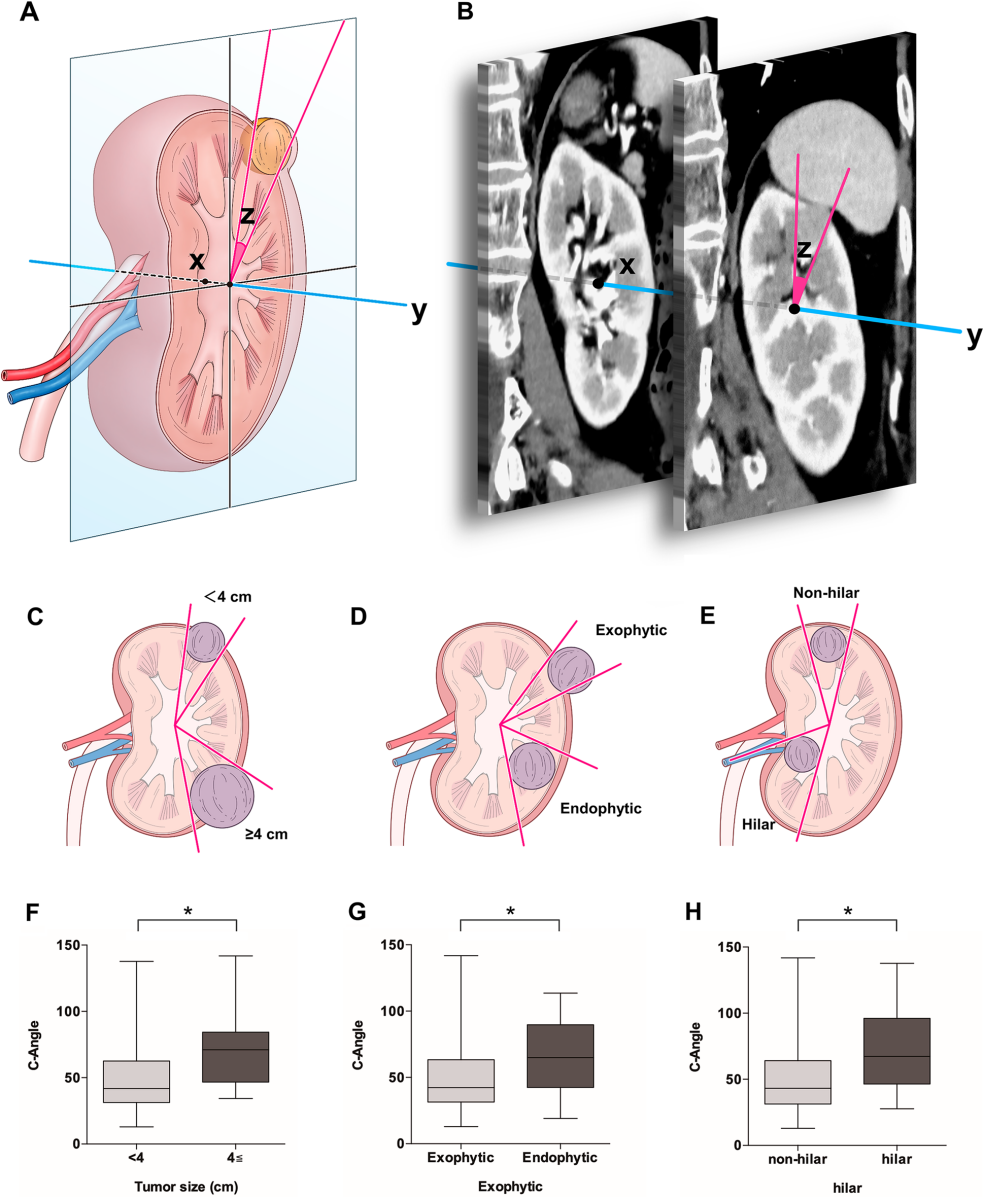
Measurement method of C-angle. (**A**) Measurement method model of C-angle. x, mid-polar reference point. y, mid-polar reference axis. C-angle was measured from the mid-polar reference axis to both outlines of the tumor invading into the kidney (z). (**B**) Measurement of C-angle using actual CT imaging. (**C**) Illustrations of concepts of size (**C**), endophyticity (**D**), and hilar tumor (**E**). Comparison of C-angle according to tumor size (**F**), endophyticity (**G**), and hilar tumor (**H**). *p < 0.001.

The C-angle measurement method required only coronal CT imaging. First, a mid-polar reference point (x) corresponding to the center of the kidney was defined using the same procedure as for the C-index^10^. The mid-polar reference axis (y) was defined as the line vertical to the coronal CT slice through the mid-polar reference point. C-angle (z) was measured from the angle from the mid-polar reference axis to both outlines of the tumor invading the kidney using the slice with the largest diameter of the tumor (Fig. 1A). In practice, the most ventral and dorsal kidney borders were identified on coronal CT imaging, and the middle slice was determined as the center slice of the most ventral and dorsal slices. In the middle slice, a mid-polar reference point was assigned to the center of an ellipse outlining the kidney. Next, CT imaging scanned the slice with the largest tumor diameter buried in the kidney and measured the C-angle from the mid polar reference axis to both outlines of the tumor (Fig. 1B).

C-angle values were measured and statistically analyzed for inter-observer reliability by 2 experienced urologists.

### Imaging protocol

All imaging was performed using a 16- or 64-slice MDCT scanner (LightSpeed or VCT, GE Healthcare). We acquired unenhanced and dynamic CT scans, including the nephrographic and excretory phases of all patients before RAPN. Coronal images were obtained at a slice thickness of 2 mm. A hilar tumor was defined as follows for the present study: (i) located entirely between the polar lines or crossing a polar line, and (ii) a maximum 5-mm distance between the tumor border and the point where the renal artery or vein enters the renal parenchyma^17^.

### Statistical analysis

Fisher’s exact test was used to compare the distributions of categorical variables. Differences in variables with a continuous distribution across dichotomous categories were tested using the Mann–Whitney U test. Univariate analysis was performed using Spearman correlation coefficients to identify associations between a continuous variable of perioperative outcomes and nephrometry scores, including C-angle, RENAL, PADUA, and C-index. Receiver operating characteristic (ROC) curves and area under the curve (AUC) were used to reveal the predictive ability of complications for nephrometry scores, as described previously^18^. The DeLong test was used to compare ROC curves. Univariate analysis of C-angle dichotomized into the C-angle < 45° group and C-angle ≥ 45° group was compared with clinical data or perioperative variables using Pearson’s chi-square and Fisher’s exact tests. Statistical significance was set at *p* < 0.05. All statistical analyses were performed using JMP Pro 14.0.0 (SAS Institute, Cary, NC, USA).

## Results

In 174 patients who underwent RAPN, the tumor was resected using the transperitoneal (n = 85) or retroperitoneal (n = 89) approach. The mean operative time was 215 min, the mean WIT 18.1 min, and the estimated blood loss (EBL) 142 ml. Forty-nine (28.2%) of the 174 patients experienced Clavien–Dindo grade I–III complications, including anemia (n = 8), pseudoaneurysm with transcatheter arterial embolization (n = 6), elevation of serum transaminase (n = 13), hematuria (n = 4), urticaria (n = 4), shoulder pain (n = 2), rhabdomyolysis (n = 2), urinary retention (n = 2), chylorrhea (n = 1), acute cholecystitis (n = 1), melena (n = 1), wound dehiscence (n = 1), abdominal incisional hernia (n = 1), and other (n = 9). Four patients received transfusion. According to the Clavien-Dindo classification, these perioperative morbidities were classified as grade 3a (n = 8), grade 2 (n = 18), and grade 1 (n = 26). Clavien-Dindo grade IV and V complications were not occured. One patient was readmitted because of pseudoaneurysm. The mean preoperative eGFR was 71.4 ml/min/1.73 m^2^, and the mean preserved eGFR was 93.5% (Table 1).

The C-Angle demonstrated no significant differences in gender (*p* = 0.384) and revealed no correlation with age in the examined population. (r = − 0.095 = 0.211) (Supplemental Fig. 1).

### C-angle was significant related to the complexity of the renal masses

The value of the C-angle is larger in the case of large tumors, endophytic tumors, and hilar renal tumors (Fig. 1C–E). When the C-angle was calculated in 174 cases, significant differences were found between tumors of < 4 cm and > 4 cm (*p* < 0.001), exophytic and endophytic tumors (*p* < 0.001), and hilar and non-hilar tumors (*p* < 0.001) (Fig. 1F–H). Together, this suggests that the C-angle is related to the complexity characteristics of the renal masses.

### C-angle and other nephrometry scores were correlated with perioperative outcomes

**Figure 2:**
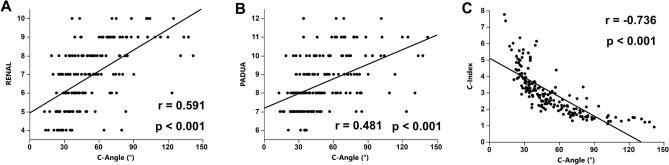
Correlation between C-angle and nephrometry scores including RENAL (r = 0.591, *p* < 0.001) (**A**), PADUA (r = 0.481, *p* < 0.001) (**B**), and C-index (r = − 0.736 *p* < 0.001) (**C**). Spearman’s correlation coefficients and p-value are indicated.

**Figure 3:**
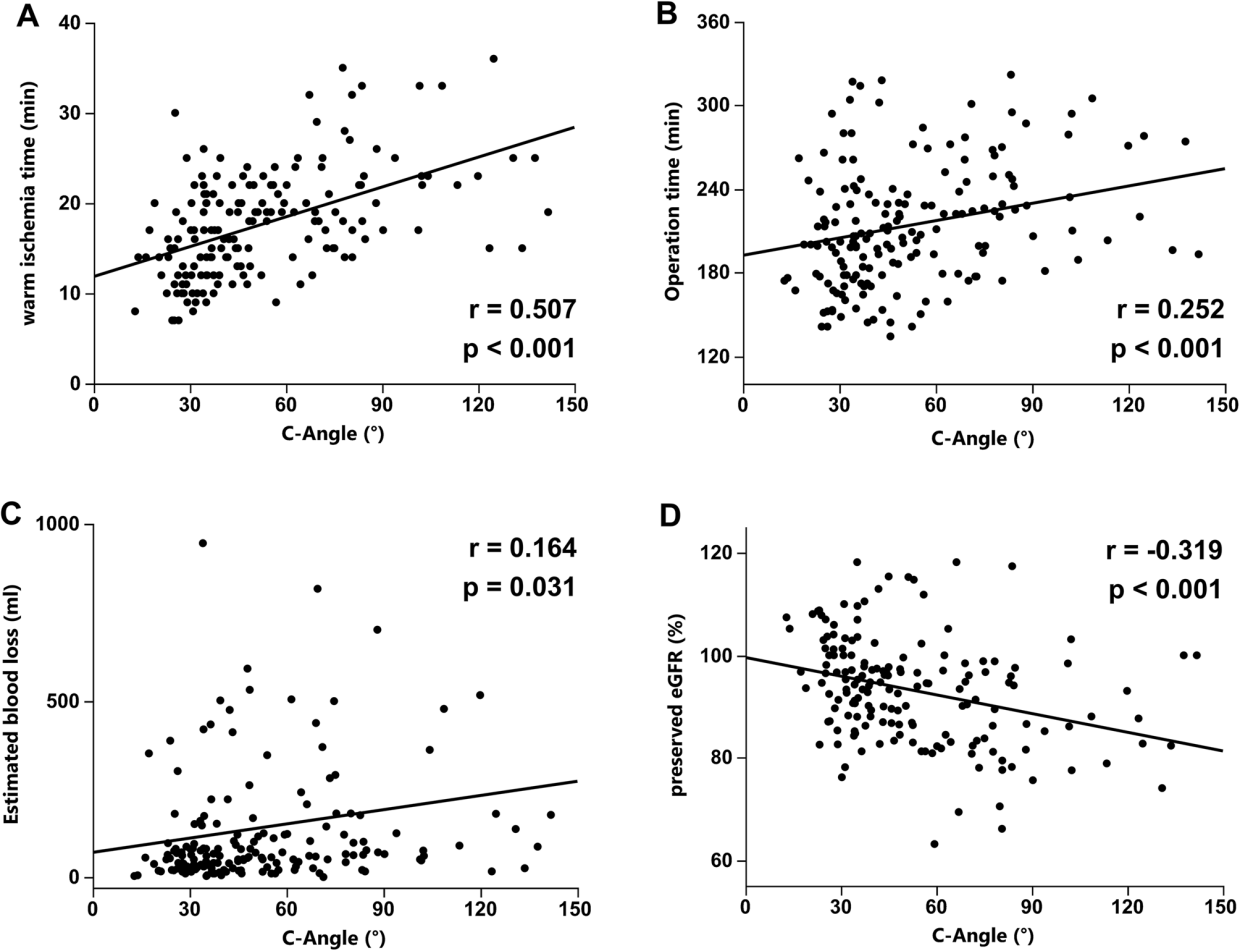
Correlation between C-angle and perioperative outcomes including warm ischemia time (r = 0.507, *p* < 0.001) (**A**), operation time (r = 0.252, *p* < 0.001) (**B**), estimated blood loss (r = 0.164, *p* = 0.031) (**C**), and preserved eGFR (r = -0.319, *p* < 0.001) (**D**). Spearman’s correlation coefficients and p-value are indicated.

To evaluate the clinical utility of C-angle as a nephrometry score for RAPN, each C-angle value was compared to existing nephrometry scores including RENAL, PADUA, and C-index. These scores are related to perioperative parameters, including warm ischemia time (WIT), operative time, EBL, preserved eGFR, and complications. In our case series of 174 patients, RENAL was significantly correlated with WIT (r = 0.4651, *p* < 0.0001) and preserved eGFR (r = − 0.251, p = 0.001), and showed no significant correlation with operative time (r = 0.145, *p* = 0.057) or EBL (r = 0.021, *p* = 0.782). PADUA was significantly correlated with WIT (r = 0.443, *p* < 0.001), operative time (r = 0.245, *p* = 0.001), and preserved eGFR (r = − 0.219, p = 0.005), but not with EBL (r = 0.099, *p* = 0.197). C-index was significantly correlated with the WIT (r = -0.426, p < 0.001), operative time (r = − 0.282, *p* < 0.001), EBL (r = − 0.198, *p* = 0.009), and preserved eGFR (r = 0.283, *p* < 0.001) (Supplemental Fig. 2). When C-angle was compared with existing nephrometry scores, significant correlations were found with RENAL (r = 0.591, *p* < 0.001) (Fig. 2A), PADUA (r = 0.481, *p* < 0.001) (Fig. 2B), and C-index (r = − 0.736, *p* < 0.001) (Fig. 2C). Next, we examined the associations between C-angle and perioperative outcomes, including WIT, operative time, EBL, preserved eGFR, and complications. Scatterplots revealed that when compared with the value of C-angle, there was a significant positive correlation with WIT (r = 0.507, *p* < 0.001) (Fig. 3A), operation time (r = 0.252, *p* < 0.001) (Fig. 3B), and EBL (r = 0.164, *p* = 0.031) (Fig. 3C), and a significant negative correlation with preserved eGFR (r = − 0.319, *p* < 0.001) (Fig. 3D).

### Comparison of predictive value of perioperative complications between C-angle and other nephrometry scores

**Figure 4:**
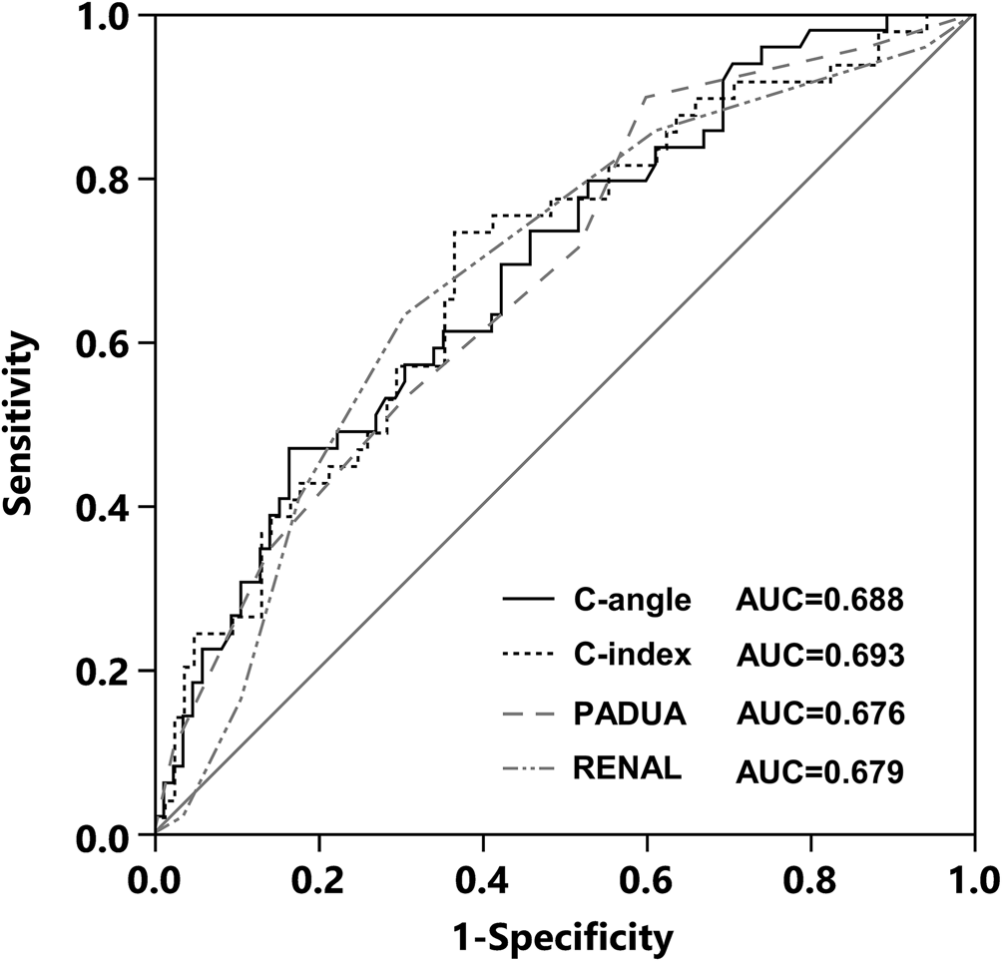
ROC curves and AUCs of C-angle, RENAL score, PADUA score, and C-index for the occurrence of perioperative complications.

To evaluate the capability of C-angle as a predictor of perioperative complications, ROC curves of C-angle for the incidence of perioperative complications were drawn and compared with those of the other nephrometry scores (Fig. 4). A C-angle cutoff value of 67.3° to predict complications was determined by ROC curve analysis, with a sensitivity of 47% and specificity of 84% in our cohort (Youden index = 0.30). The AUC value for C-angle in predicting perioperative complications was 0.688 (95% CI 0.590–0.772). The AUC value showed no significant difference between C-angle and the other nephrometry scores, including RENAL (AUC = 0.676, 95% CI 0.579–0.761, *p* = 0.757), PADUA (AUC = 0.679, 95% CI 0.580–0.764, *p* = 0.837), and C-index (AUC = 0.693, 95% CI 0.593–0.777, *p* = 0.847) (Table 2).

**Table 2:** Predictive value of perioperative complications between C-angle and other nephrometry scores.

	AUC	95% CI	Sensitivity	Specificity	Cut-off	Youden index	*p*-value
Complications: Clavien–Dindo classification I–III							
C-angle	0.688	0.590–0.772	0.47	0.84	67.3	0.30	
RENAL	0.676	0.579–0.761	0.90	0.40	6	0.30	0.757
PADUA	0.679	0.580–0.764	0.63	0.69	9	0.33	0.837
C-index	0.693	0.593–0.777	0.74	0.64	2.84	0.37	0.847

### Inter-observer agreement for C-angle

**Figure 5:**
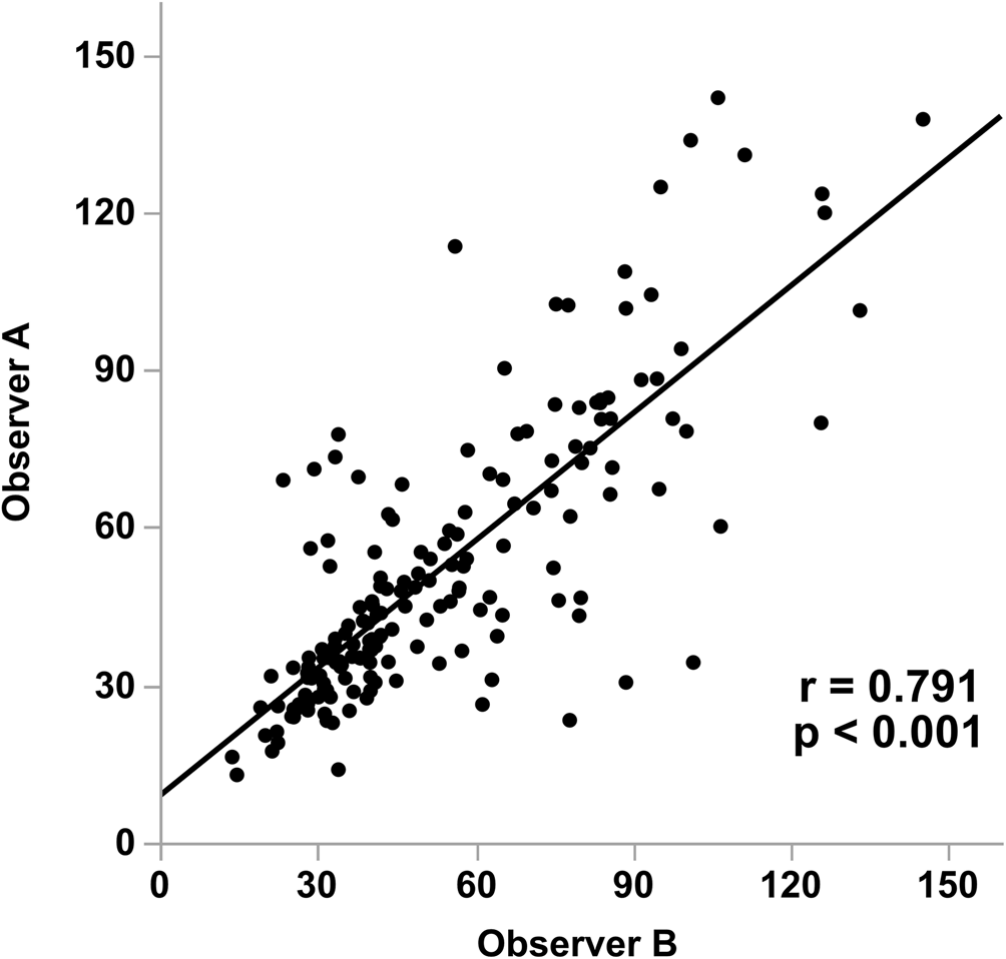
Correlation between Observer A and B for C-angle.

To evaluate the reproducibility of the C-angle, we compared the C-angles of the two observers in a scatterplot. C-angle had a strong correlation between the two observers. (r = 0.791 *p* < 0.001) (Fig. 5).

### C-angle as a representation of renal tumor complexity and association with perioperative outcomes and other nephrometry scores

To evaluate whether C-angle could represent the clinical characteristics of renal tumors, all 174 patients were dichotomized according to a 45° C-angle. Despite the lack of significant differences in patient demographics between the two groups, significant differences were found in tumor size (*p* < 0.001), clinical T1b (*p* = 0.004), endophytic status (*p* = 0.034), and hilar tumor (*p* = 0.004). Also, relationships between the higher C-angle group and higher RENAL score (*p* < 0.001), higher PADUA score (*p* < 0.001), and lower C-index (*p* < 0.001) were observed. No significant associations were found between C-angle and pathological outcomes. Patients in the greater than 45° C-angle group had worse perioperative outcomes, including longer operative time (*p* = 0.010), longer WIT (*p* < 0.001), and other complications (*p* = 0.028). Regarding preserved renal function, a greater reduction in eGFR was found for C-angle greater than 45° (*p* < 0.001). In conjunction with these results, C-angle was able to represent the complexity of the renal tumor and was associated with a worse perioperative outcome and lower rate of residual renal function after RAPN (Table 3).

**Table 3:** Association between C-angle and patient demographics, tumor characteristics, and pathological and perioperative outcomes.

	All	C-angle < 45°	C-angle ≥ 45°	*p* Value
	n = 174	n = 87	n = 87	
Patient demographic				
Age, years mean (range)	62 (26–88)	63 (35–88)	61 (26–84)	0.083
Body mass index, kg/m^2^, mean ± SD	24.2 ± 4.3	24.0 ± 3.6	24.4 ± 5.0	0.552
Sex, n (%)				
Male	127 (73.0)	63 (72.4)	64 (73.6)	0.864
Female	47 (27.0)	24 (27.6)	23 (26.4)	
Tumor characteristics				
Kidney, laterality, n (%)				
Right	93 (53.5)	50 (57.5)	43 (49.4)	0.287
Left	81 (46.5)	37 (42.5)	44 (50.6)	
Tumor size, mm, mean ± SD	26.5 ± 10.5	22 ± 8.7	31 ± 10.3	< 0.001
Clinical T stage, cT1a, n (%)				0.004
T1a	151 (86.8)	82 (94.3)	69 (79.3)	
T1b	23 (13.2)	5 (5.7)	18 (20.7)	
Endophytic depth, n (%)				0.034
< 50% endophytic	56 (32.2)	35 (40.2)	21 (24.1)	
50–100% endophytic	92 (52.9)	44 (50.6)	48 (55.2)	
100% endophytic	26 (14.9)	8 (9.2)	18 (20.7)	
Hilar tumor, n (%)				0.004
Hilar	28 (23.2)	7 (8.0)	21 (24.1)	
Non-hilar	146 (76.8)	80 (92.0)	66 (75.9)	
Nephrometry index				
C-angle, °, mean ± SD	52.4 ± 27.2	31.9 ± 7.2	72.9 ± 24.1	< 0.001
C-index score, mean ± SD	3.05 ± 1.44	4.03 ± 1.36	2.07 ± 0.63	< 0.001
RENAL score, median (range)	7 (4–10)	5 (4–10)	8 (4–10)	< 0.001
PADUA score, median (range)	8 (6–12)	7 (6–12)	9 (7–12)	< 0.001
Pathological outcome				
Malignant	163 (93.7)	80 (92.0)	83 (95.4)	0.350
Clear	134 (77.0)	68 (78.2)	66 (75.9)	0.360
Non-clear	29 (21.6)	12 (13.8)	17 (19.5)	
Non-malignant	11 (6.3)	7 (8.0)	4 (4.6)	
Perioperative outcome				
Operative time, min, mean ± SD	215.5 ± 48.2	206.0 ± 47.3	225.0 ± 49.2	0.010
Warm ischemia time, min, mean ± SD	18.1 ± 7.4	14.9 ± 4.8	21.2 ± 9.4	< 0.001
Estimated blood loss, ml, mean ± SD	142 ± 223	114 ± 202	170 ± 242	0.102
Complications: Clavien–Dindo I-III, (–)	49 (28.2)	18 (20.7)	31 (35.6)	0.028
Complications: Clavien–Dindo III, (%)	8 (4.6)	3 (3.5)	5 (5.8)	0.469
Renal function				
Preoperative eGFR, ml/min/1.73 m^2^, mean ± SD	71.4 ± 16.5	71.3 ± 16.1	71.7 ± 17.2	0.889
Preserved eGFR (6-mo/pre), %, mean ± SD	93.5 ± 10.4	96.1 ± 8.5	90.2 ± 11.6	< 0.001

## Discussion

In this study, we showed the usefulness of the C-angle, as calculated from CT images, to evaluate the complexity of renal tumors and predict perioperative outcomes. Preoperative evaluation using nephrometry scores, such as RENAL or PADUA, is generally recommended to predict the potential morbidity of NSS^3^. However, these scoring systems have limitations such as insufficient interobserver reproducibility and incomplete quantification of relevant anatomical features. In addition, overlaps exist between some features of nephrometry score parameters that may complicate preoperative evaluation. Consequently, the Simplified PADUA REnal (SPARE) nephrometry system, which excluded polar location and urinary collecting system involvement, was suggested and had similar predictive accuracy to the original PADUA score^19^. Likewise, the existing mathematical-based nephrometry scores might be complicated for clinical use because they require three-dimensional construction, very thin-slice CT imaging, or structural assumptions. Therefore, a simplified mathematical assessment-based nephrometry score might also be useful.

C-angle is simply the angle a renal tumor occupies from the center of the kidney and can be measured using only coronal CT imaging without complex calculations. Considering the anatomical features and surgery of the kidney, C-angle is reasonably accessible in terms of the radial anatomical architecture and surgical approach for partial nephrectomy, because the incision is usually performed vertically from the surface along an anatomical architecture^20^.

Because significant associations between C-angle and other existing nephrometry scores were found, C-angle could involve the components of RENAL, PADUA, and C-index. In the present study, C-angle might be useful as a nephrometry score because it correlated with WIT, operative time, EBL, residual renal function, and perioperative complications, as well as with other nephrometry scores.

In our cohort, we observed a high inter-observer reliability in the C-angle. C-angle may serve as a nephrometry score with high reproducibility.

When perioperative outcomes were compared by dichotomizing C-angle into 45° groups, patients with tumors > 45° had worse perioperative outcomes, such as longer operative time, longer WIT, increased complications, and decreased renal function. Therefore, the simple nephrometry score expressed by C-angle was sufficiently predictive of perioperative outcomes and might be an indicator of technical difficulty for RAPN.

This study had several limitations. First, this was a retrospective cohort with relatively few cases, and the outcomes should be validated in a larger cohort. In addition, C-angle could not be measured in three cases because it overlapped with the axial polar axis. In such cases, measurement may be possible using axial CT imaging. Furthermore, this research is an initial report and has not been externally verified to ensure the objectivity of C-angle. It will be necessary to increase the reliability of C-angle through external verification. Finally, C-angle represents only some aspects of renal tumor complexity and lacks geographical location information for the kidney. For accurate evaluation of tumor complexity, C-angle should be combined with original CT imaging or other nephrometry scores.

## Conclusion

C-angle might be a suitable criterion for evaluating the complexity of renal tumors. This novel nephrometry score is intuitive and can easily be measured using CT imaging alone. C-angle can predict significant perioperative outcomes, including operative time, WIT, and preserved renal function, as effectively as other existing nephrometry scores in our cohort. Further validation in larger cohorts will be needed to determine whether C-angle could be used for decision-making in the treatment of patients or to guide surgical planning of NSS.

### Supplementary Information

Supplementary Figure 1.

Supplementary Figure 2.

## Data Availability

The dataset analyzed during this study are not publicly available because of individual privacy. They are available from the corresponding author on reasonable request.
