# Supplementary material for: Centrality angle is a novel nephrometry score to predict tumor complexity and perioperative outcomes for partial nephrectomy
Source: Sci Rep. 2024 Feb 27;14:4780. doi: 10.1038/s41598-024-55448-0 (PMC10899191; doi:10.1038/s41598-024-55448-0)
Supplement: Supplementary file 1 — Supplementary Figure 1. [file 41598_2024_55448_MOESM1_ESM.pptx]

## Slide 1
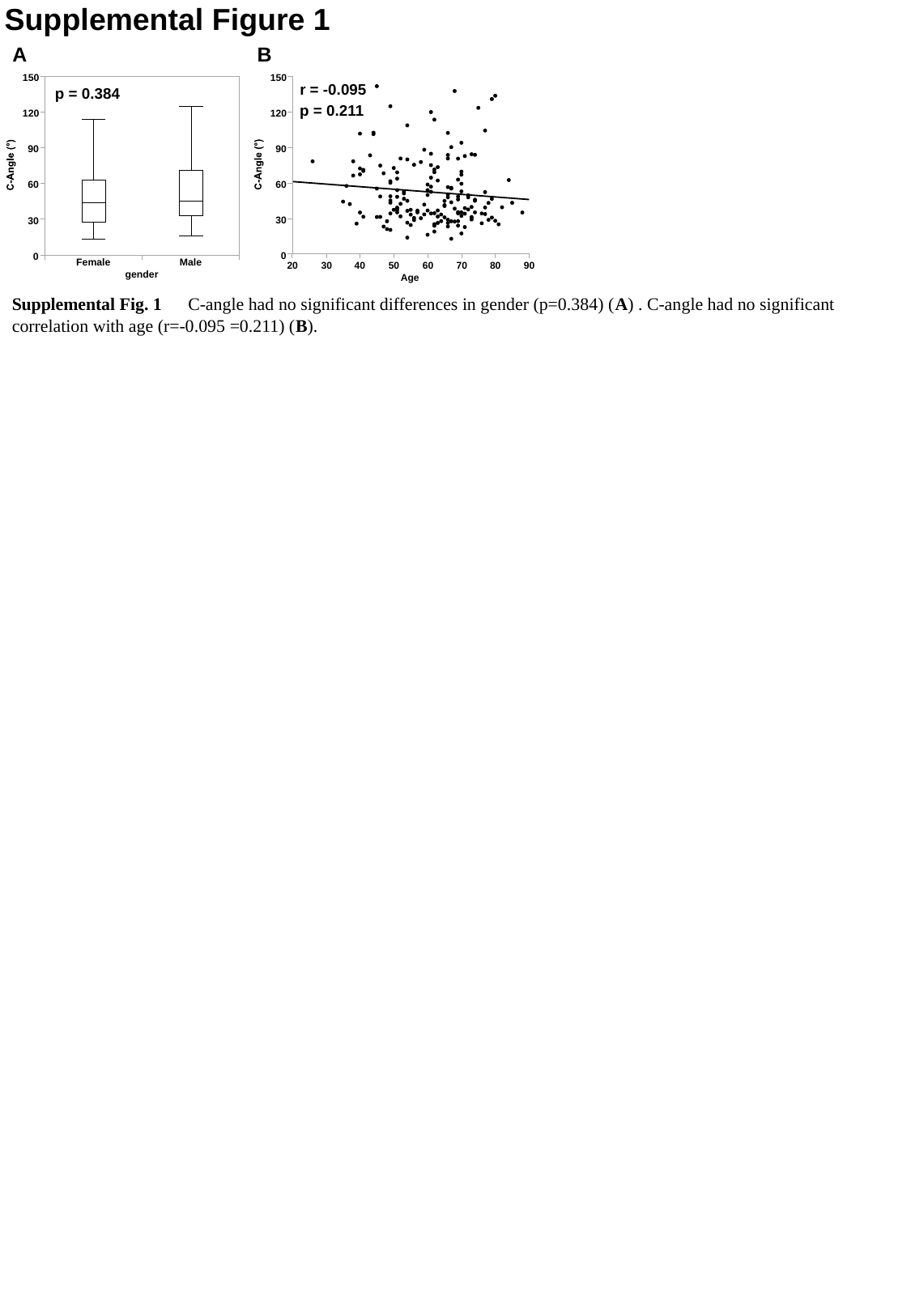

Supplemental Figure 1
A
B
r = -0.095
p = 0.384
p = 0.211
Supplemental Fig. 1　C-angle had no significant differences in gender (p=0.384) (A) . C-angle had no significant correlation with age (r=-0.095 =0.211) (B).
