# Supplementary material for: Centrality angle is a novel nephrometry score to predict tumor complexity and perioperative outcomes for partial nephrectomy
Source: Sci Rep. 2024 Feb 27;14:4780. doi: 10.1038/s41598-024-55448-0 (PMC10899191; doi:10.1038/s41598-024-55448-0)
Supplement: Supplementary file 2 — Supplementary Figure 2. [file 41598_2024_55448_MOESM2_ESM.pptx]

## Slide 1
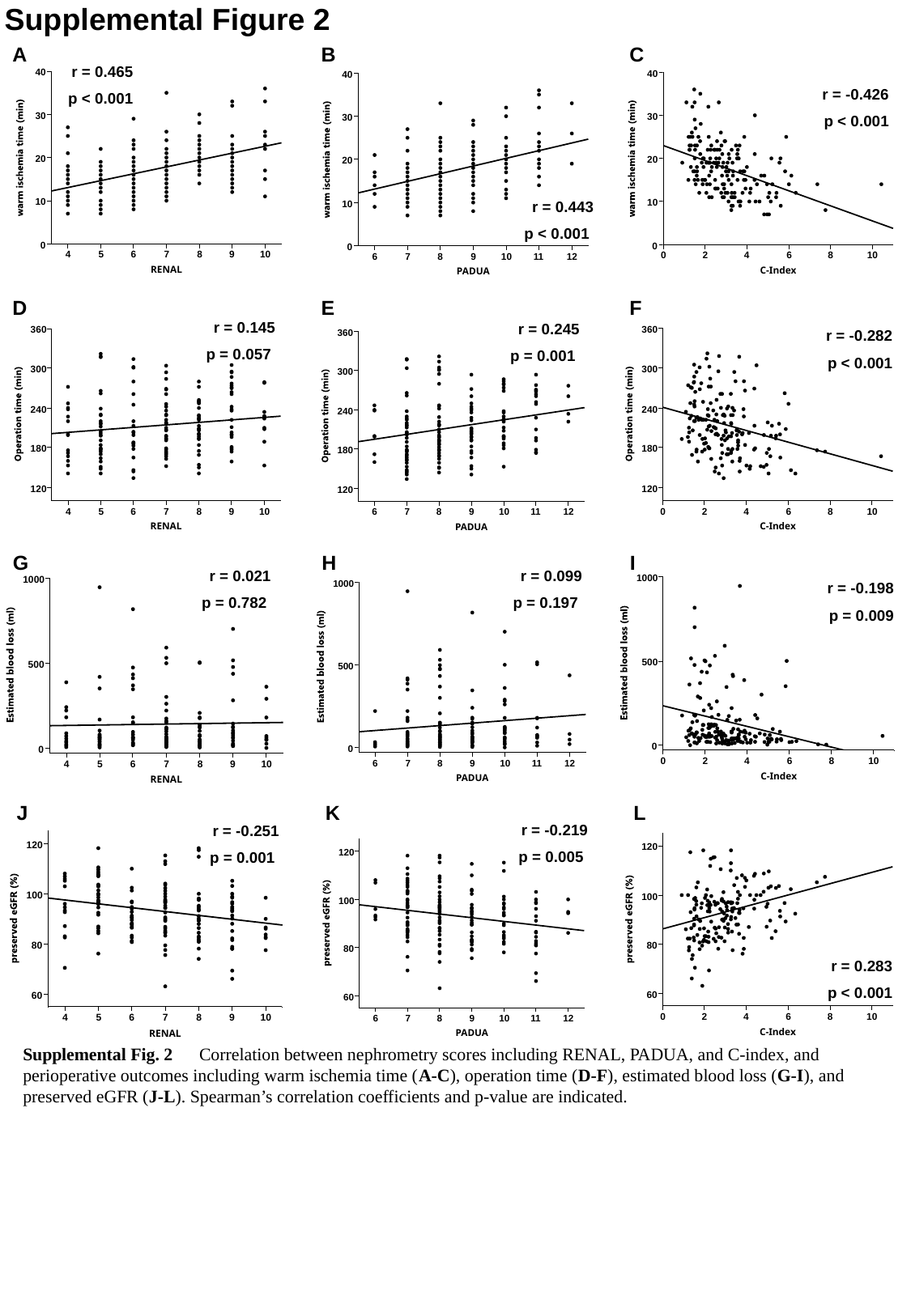

Supplemental Figure 2
C
A
B
r = 0.465
r = -0.426
p < 0.001
p < 0.001
r = 0.443
p < 0.001
F
D
E
r = 0.145
r = 0.245
r = -0.282
p = 0.057
p = 0.001
p < 0.001
I
G
H
r = 0.099
r = 0.021
r = -0.198
p = 0.197
p = 0.782
p = 0.009
L
J
K
r = -0.219
r = -0.251
p = 0.005
p = 0.001
r = 0.283
p < 0.001
Supplemental Fig. 2　Correlation between nephrometry scores including RENAL, PADUA, and C-index, and perioperative outcomes including warm ischemia time (A-C), operation time (D-F), estimated blood loss (G-I), and preserved eGFR (J-L). Spearman’s correlation coefficients and p-value are indicated.
